# An overview of sodium-ion batteries as next-generation sustainable electrochemical devices beyond the traditional lithium-ion framework

**DOI:** 10.55730/1300-0527.3707

**Published:** 2024-09-17

**Authors:** Gamzenur ÖZSİN

**Affiliations:** Department of Chemical Engineering, Faculty of Engineering, Bilecik Şeyh Edebali University, Bilecik, Turkiye

**Keywords:** Sodium-ion battery, electrochemical energy storage, battery, electrode materials, electrolyte

## Abstract

The rise in the popularity of electric vehicles and portable devices has boosted the demand for rechargeable batteries, with lithium-ion (Li-ion) batteries favored for their superior energy and power density. However, supply strains and sustainability issues are driving the search for alternatives. Postlithium technologies, particularly sodium-ion (Na-ion) batteries, are gaining attention for their promising potential and similarity to Li-ion technology. While efforts are still needed to enhance the energy and power density as well as the cycle life of Na-ion batteries to replace Li-ion batteries, these energy storage devices present significant advantages in terms of sustainability, theoretical capacity, and intrinsic safety features. Through this paper, the current state of Na-ion batteries, focusing on key components such as anodes, electrolytes, cathodes, binders, separators, and current collectors, has been critically assessed. Recent advancements, challenges, future directions, and new materials engineering strategies for improving electrochemical performance are also discussed. Overall, this review offers a comprehensive analysis of the development of high-performance, cost-effective, and sustainable energy storage systems.

## 1. Introduction

Developing sustainable energy production methods is essential for a circular economy, particularly considering growing concerns over the consequences of fossil fuels. To meet the significant problems of climate change, resource depletion, and rising urbanization in the upcoming years, collaborative efforts are needed to develop a sustainable and low-emission energy model. This energy transformation will rely heavily on the widespread utilization of renewable and eco-friendly sources which are unfortunately sporadic and influenced by weather patterns and seasonal variations [1]. Even though a clean and fully renewable energy system that is independent of fossil resources does not seem probable in the very near future, policymakers must urgently move in the direction of sustainable solutions to pass on a cleaner planet to future generations.

Rechargeable battery technologies stand out as the most popular energy storage technologies across diverse locations due to their versatility in terms of power and energy density, efficiency, weight, and system mobility. The renewable energy source can be stored in battery packs; for instance, their contribution to wind and solar energy storage can be considered a crucial and significant step in reducing dependency on fossil fuels. With their use in several different industries, such as in electric or hybrid automobiles, renewable energy systems, autonomous and robotic systems, battery storage technologies, grids, and the development of green energy strategies have received increasing attention [2–4]. In particular, industries for electric vehicles (EVs) will require much more efficient electrochemical energy storage soon. It is estimated that 57% of all passenger vehicle sales will be electrified by 2040. Based on the International Energy Agency’s Net Zero 2050 Roadmap, it is projected that EVs will comprise 20% of the automotive market by 2030 and increase to 86% by 2050. This will result in an immense opportunity according to the Economic Transition Scenario, with a market potential of $1.9 trillion for charging infrastructure between now and 2050. Currently, Li-ion batteries are the mainstream technology for EV batteries owing to their superior energy-to-weight ratio. On the other hand, the increasing demand for minerals such as lithium, cobalt, and manganese, essential components of Li-ion batteries, is driving up both their demand and market prices in an irrepressible way. Based on certain forecasts,^1^,^2^ it is anticipated that a minimum of one newly operational lithium mine will be requisite annually until 2025 to fulfill the projected surge in lithium demand. Lithium has been deliberately stocked since it is a resource that is becoming more strategically important and because production has been outpacing consumption. This led to lithium resources reaching 80 million tons, while reserves stood at only 17 million tons in 2020, followed by a six-fold increase in the mineral’s price during the same period [4]. Despite the notable rise in production and prices, significant technological advancements have not materialized, constraining the utilization of the doubled lithium resources acquired over the past 5 years. Unfortunately, this limitation has contributed to the escalating prices as well. Furthermore, the Li supply chain comes with political and supply risks that may affect the sustainability of energy storage in future. According to the Mineral Commodity Summaries in 2022, ensuring the security of lithium supply has been identified as a paramount concern for technology companies [5]. To address this, strategic collaborations and partnerships between technology firms and exploration entities continue to be forged intensely. These alliances aim to guarantee a dependable and diversified lithium supply for both battery suppliers and vehicle manufacturers.

**Figure 1:**
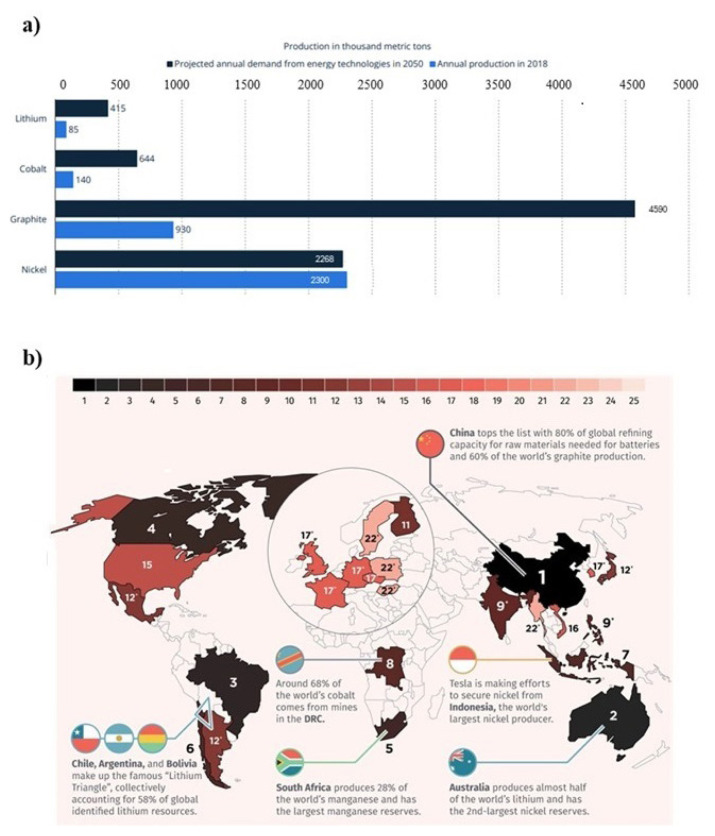
(a) Global production of battery raw materials in 2018 with forecasted demand stemming from energy technologies by 2050^1^ (categorized by mineral and expressed in 1000 metric tons) and (b) EV battery supply chain in 2020.^2^

In addition to Li; Co, Ni, and graphite used in Li-ion batteries are among the critical minerals that may be further threatening energy security and industrial resilience whose global demand is projected to increase by between 6- and 13-fold by 2040 [6,7]. Figure 1 shows the key materials needed for Li-ion batteries and the status of the fragile EV battery supply chain.^3^,^4^

Therefore, deeper scientific investigations into novel energy storage mechanisms that surpass conventional Li-ion technology, such as lithium-air, lithium-sulfur, magnesium, and sodium-ion batteries, has captivated the attention of researchers towards exploring innovative materials for these technologies. The global paramount concerns about sustainable energy technologies and the growing use of batteries have become therefore a critical aspect for technological evolution. At this point, sodium-ion (Na-ion) technologies have become attractive because they present a range of potential benefits in comparison to Li-ion batteries in terms of sustainability, notwithstanding the nascent stage of their technological advancement, which necessitates further development and may result in a protracted period before commercial viability is achieved.

### 1.1. A brief overview on Na-ion batteries

**Figure 2:**
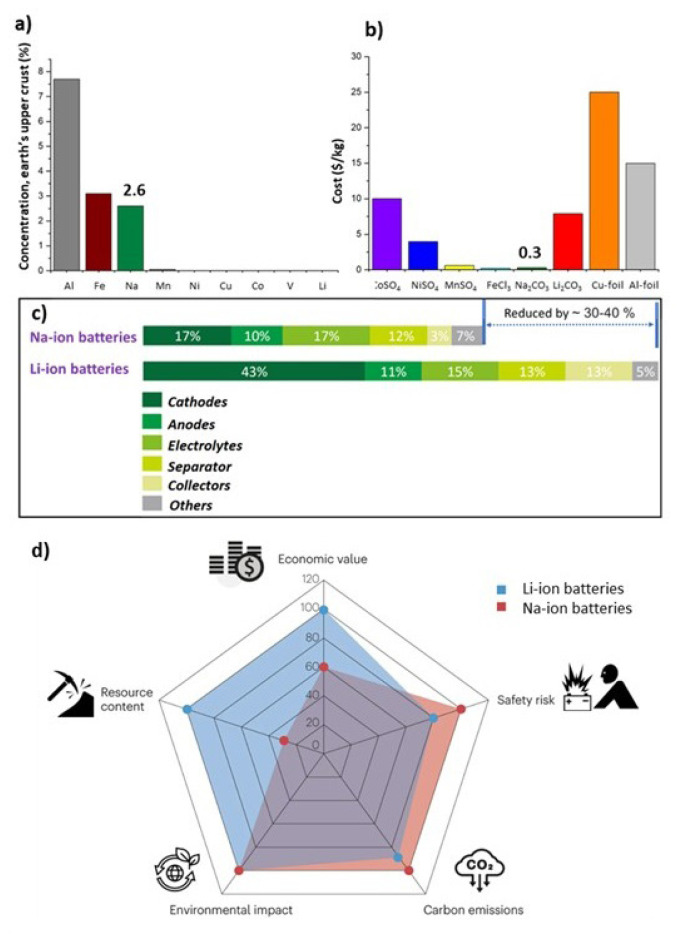
(a) The prevalence of elements in the Earth’s upper crust commonly used in batteries, (b) pricing of the raw materials for cathodes and the current collector foils-$/kg [14], (c) cost comparison among the materials of Na- and Li-ion batteries [15], and (d) a radar chart to compare various aspects of Na- and Li-ion batteries [16]. (Figure reprinted with permission.)

**Figure 3:**
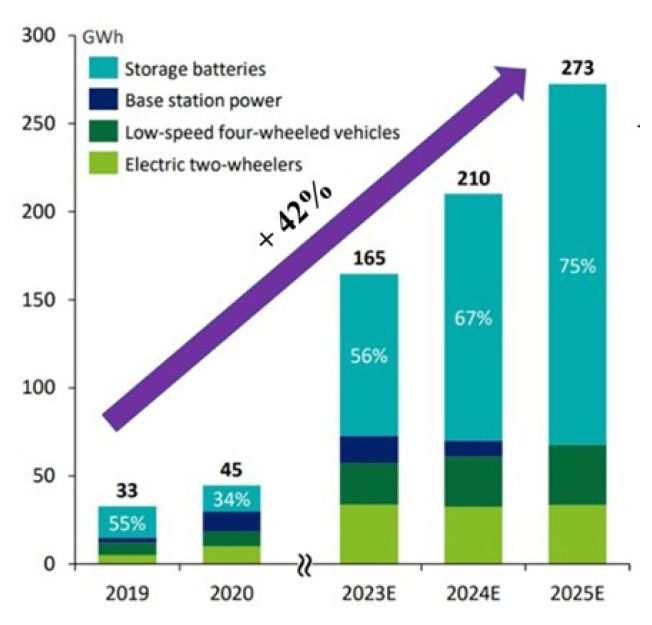
Installed capacity projection of Na-ion battery by potential application [16]. (Figure reprinted with permission.)

Na-ion batteries have been seen alongside lithium cells since the early 1970s, according to a review of the literature. Na-ion battery development was abandoned for a period when most scientists began focusing on Li-ion batteries after 1991. However, research on Na-ion batteries was not completely discontinued; during that period, efforts were made to find a potential anode material. Based on an unequal distribution of resources for lithium, research efforts have intensified to explore nonlithium insertion materials. Surprisingly, since 2010, there has been a notable rise in the number of scientific publications focusing on Na-ion batteries. Scientists have initiated a re-evaluation of the previously disregarded Na-ion battery technology, examining its potential as a complementary technology to Li-ion batteries over the last decade. This shift is driven by growing apprehension regarding the sustainability of lithium sources and their uneven distribution [8]. In a recent study by Au et al., full-spectrum sustainability of batteries was critically analyzed [9]. More emphasis is placed on the necessity of establishing international regulations, coordinating efforts across all stakeholders involved in the battery supply chain, and conducting comprehensive cradle-to-cradle assessments encompassing cost, performance, and environmental impacts. These measures are deemed essential for facilitating the shift towards a genuinely sustainable energy market. Figure 2 illustrates the abundance of elements found in the earth’s upper crust, focusing on those frequently utilized in batteries, alongside the pricing information for raw materials pertaining to cathodes and current collector foils. Considering both the economic and geopolitical distribution of Li-ion battery components, Na-ion technologies show significant advantages for the next-generation energy storage technologies. As can be seen from the figure, sodium, as an alternative alkali metal, is widely available and equally distributed across the globe. Its widespread availability mitigates the likelihood of tensions stemming from competition and price escalation. With sodium exhibiting greater availability in terms of both volume and geographic distribution compared to lithium, sodium carbonate prices remained below $1 per kilogram in 2019, while lithium was priced at $13 per kilogram. This tremendous abundance and the widespread geographical distribution of Na compared with Li should theoretically make the mineral more resistant to any pricing fluctuations caused by political or economic disruptions. As a result, it is accepted that the combination of decreased costs and increased availability of sodium renders Na-ion batteries an appealing substitute for Li-ion batteries [10]. As can be seen from Figure 3, projections about Na-ion batteries have likely been more promising mainly due to the improvements in their performance, including cycle life, efficiency, and safety. In particular, the radar chart illustrates resource, safety, economic value, carbon emissions, and environmental impact for 1 kg of spent Li- and Na-ion batteries objectively. Given their potential as appealing supplementary energy sources, despite the obstacles still present in the commercialization phase, Na-ion batteries show promise for developing nations seeking to adopt energy storage solutions soon. The economic evaluation by Bai and Song using the bottom-up assessment framework indicated that Na-ion batteries currently fall short in terms of competitiveness when compared to other technologies examined in their analysis. This is attributed to their shorter lifespan and higher costs. However, it is noteworthy that they boast the smallest environmental impact and the shortest payback period. This indicates their considerable potential to soon become the most effective and environmentally friendly energy storage option [11]. According to the analysis by Hirsh et al. [12], transition from lithium- to sodium-driven technologies should be supported by industry investments to facilitate extensive testing and evaluation at the cell/pack level, akin to the successful trajectory of Li-ion batteries over the past four decades. Undeniably Na-ion batteries are the most promising option for enhancing the resilience of our electrical grid, promoting renewable energy storage and distribution, and reducing our dependence on traditional fossil fuels since they involve cost-efficient raw materials and innovative cell designs [12,13].

**Figure 4:**
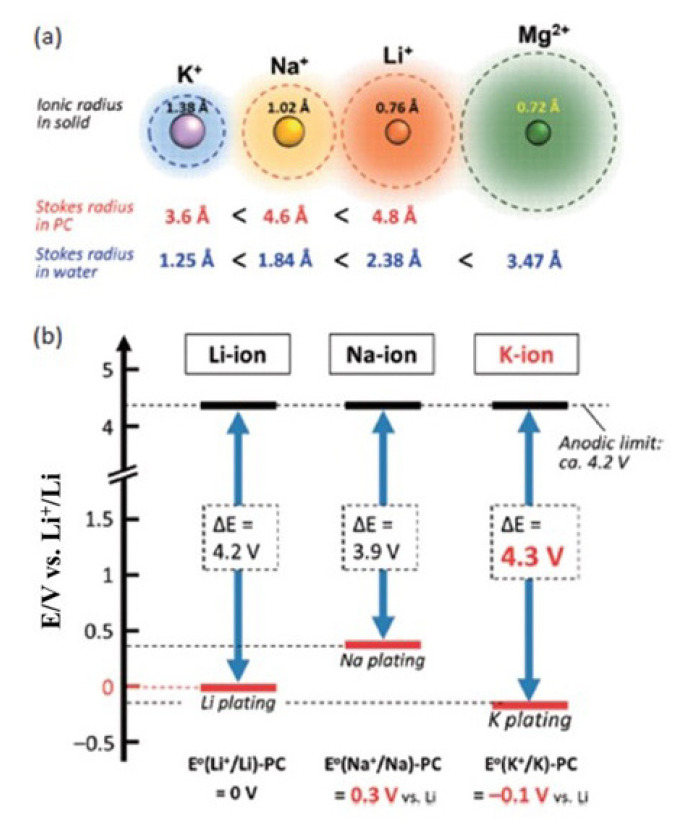
Comparing (a) the ionic size in solid form and the Stokes radii in water and propylene carbonate solutions among Li^+^, Na^+^, K^+^, and Mg^2+^ ions, and (b) assessing the potential window of alkali-metal ion batteries, considering nonaqueous electrolyte solutions with carbonate ester solvents [8]. (Figure reprinted with permission.)

Although Na-ion and Li-ion batteries share a common working principle, Na-ion batteries exhibit lower energy density and slower reaction kinetics. This is attributed to sodium’s higher redox potential of −2.71 V vs. the standard hydrogen electrode (SHE) for Na^+^/Na, its heavier ion with a molecular weight of 23 g/mol, and its larger ionic radius of 1.02 Å compared to lithium (with a redox potential of −3.01 V, a molecular weight of 6.9 g/mol, and an ionic radius of 0.76 Å). The standard electrode potential, crystal ion size, and atomic weight of sodium and lithium are compared in Figure 4. Consequently, substituting lithium ions (Li^+^) with sodium ions (Na^+^) inevitably leads to a reduction in voltage and capacity, despite the occurrence of similar redox reactions at the positive and negative electrodes [14–17]. The transport characteristics of alkali-metal ions within electrolytes, such as ionic conductivity and transference number, typically impact the rate performance. Given that Na^+^ ions possess weaker Lewis acidity compared to Li^+^, they exhibit smaller Stokes’ radii across various solvents as a consequence [18].

**Figure 5:**
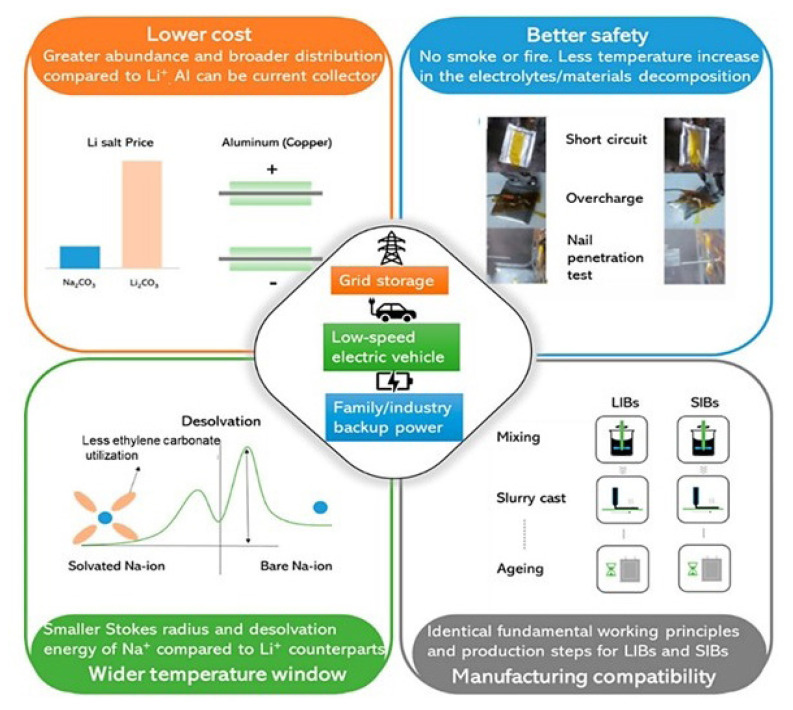
Schematic demonstration of Na-ion batteries’ main benefits [23]. (Figure reprinted with permission.)

Arguably one of the biggest advantages of Na-ion batteries is that Al may be used as a negative current collector instead of Cu [19] while Li alloys with Al. It is important to note that Al is not only more affordable and lightweight than Cu, but it also makes the battery safer by serving as a negative current collector. If Li-ion batteries are overdischarged and the Cu current collector potential rises above approximately 3.5 V vs. Li^+^/Li, Cu will oxidize and begin to dissolve into the electrolyte. During subsequent charging of the battery, dendrites may form as the dissolved Cu is reduced onto the anode, potentially leading to short circuits and hence posing risks of hazardous flames or explosions [20,21]. On the other hand, Al is passivated by the electrolyte and does not dissolve even when charged to voltages greater than 4 V vs. Li^+^/Li. The superior stability of the Al negative current collector can even allow the Na-ion battery to be discharged to an operating voltage of 0 V (V_cathode_ = V_anode_ ≈ 2.8 V vs. Na^+^/Na) for dramatically safer transportation, as recently demonstrated by the start-up company Faradion. Another benefit of the Na-ion chemistry is the option to utilize a greater variety of elements (including cheap, abundant Fe) in layered NaMO_2_ intercalation cathodes. The layered structure that develops in these types of compounds, due to significant differences in ionic radii between small transition metal ions and larger alkali metal ions, results in compositional diversity [22]. Figure 5 illustrates the main benefits of Na-ion batteries, including lower cost, enhanced safety, better temperature performance, and compatibility with Li-ion technologies, positioning them as a well-suited option for large-scale electrochemical storage applications in renewable energy systems [23]. Overall, Na-ion batteries possess certain advantages from a safety and structural perspective. Although the economic benefit of Na-ion batteries may have been slightly exaggerated in the past, the low cost advantage could still be realized if electrode materials with higher energy density and longer cycle life are developed. The importance of discovering novel materials and chemicals has increased in response to market demands for high energy density, low cost, and enhanced safety. For accomplishing this aim, Na-ion batteries have received increasing consideration as a technology beyond Li-ion chemistry. Notably, current Li-ion battery production lines and available equipment can be easily adapted for Na-ion battery manufacturing [23,24]. As sodium is the second lightest and second-smallest alkali metal together with cost and safety benefits, Na-ion batteries can solve primary concerns without sacrificing energy density, if efficient electrochemical Na insertion/deinsertion is accomplished. To address the confluence of challenges posed by environmental pollution, energy scarcity, and economic considerations, Na-ion batteries have emerged as a pivotal solution for advancing sustainable energy storage technologies. Hence, the discovery of innovative active materials and electrolytes has the potential for achieving substantial enhancements in the performance of Na-ion batteries.

## 2. Basic operation principle of Na-ion batteries

Simply put, batteries store chemical energy, convert it into electrical energy, and use it. If this transformation is one-way, the battery is a primary system, which cannot be charged. On the other hand, if the conversion can take place in either direction, the battery is a secondary system with rechargeable features, and hence it is called secondary. Rechargeable batteries, which are included in battery technology, have been used in these systems due to their advantages, such as high energy capacity, high storage efficiency, and fast response time. The battery is an incredibly efficient transducer of energy from chemical to electrical form and vice versa. It operates by separating the two half reactions of a reduction/oxidation (redox) reaction. When the battery is discharged, the anode is oxidized (donates electrons) and the cathode is reduced (receives electrons). To maintain charge neutrality, ions also flow proportionally from the anode to the cathode, and this process occurs reversibly during charging. Because the electrolyte only conducts ions between the two half reactions, the redox reaction can only proceed if the electrons journey out of the battery and around an outer circuit. The internal chemical reaction of the battery can therefore be governed by controlling the flow of electrons on the external circuit. This can be achieved using something as complicated as a battery management system or as simple as a light switch.

**Figure 6:**
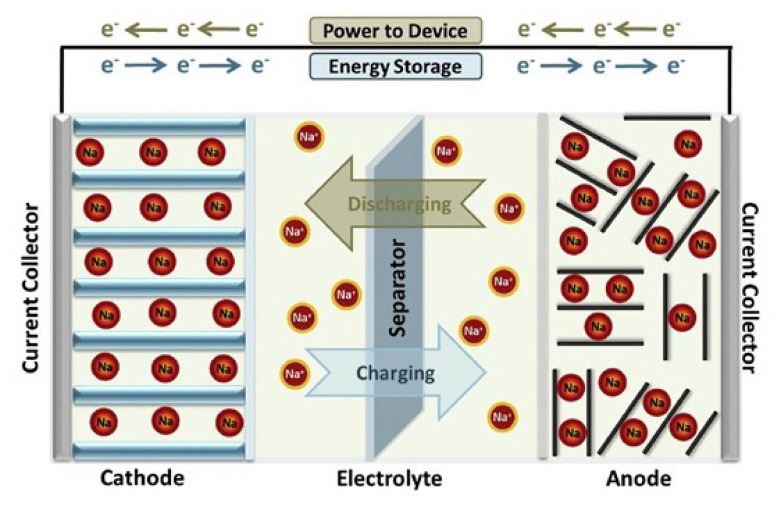
Schematic representation for the Na-ion battery operation [26]. (Figure reprinted with permission.)

The principle of Na-ion batteries is the same as that of Li-ion batteries, since the cathode active material, as a positive electrode, releases electrons into the external circuit during charging, leading to the oxidation of transition metal ions. To preserve charge neutrality, some of the intercalated sodium atoms dissolve into the electrolyte as ions in Na-ion batteries. They move towards the negative electrode (anode), where they are integrated into the structure to restore the disrupted charge neutrality caused by the electrons transferred from and received by the cathode. The process is reversed during discharge. This entire sequence of reactions takes place within a closed system, where for every electron generated during oxidation, an electron is consumed in the reduction reaction at the opposite electrode [25]. The operating principle of Na-ion batteries is shown in Figure 6.

The Na-ion battery consists of three main components: the cathode (oxidant), anode (reductant), and electrolyte. The current flowing across the cathode and anode is gathered by Al/Cu current collector foils, which then supply it to the redox centers of the electrodes and the porous separator, functioning as an ion filter to prevent reaction between the electrodes. During charging, Na^+^ ions migrate from cathode to anode through the electrolyte, while electrons traverse the external circuit to the anode, where they are collected by the Al current collector. However, during discharge, electron and ion transport occurs in opposite directions. Electrons must not traverse the electrolyte to prevent internal short-circuiting through electrolyte decomposition. Crucially, the electrochemical cell must exhibit reversible reactions at the electrodes [26].

## 3. Main components of Na-ion batteries

The desired characteristics of basic battery components have been summarized in Table 1. The subtle point to consider for a suitable design of a Na-ion battery is a comprehensive understanding of Na^+^ ion insertion. Ion insertion into an electrode can be delineated through various common phases, including electronic conduction across an active material layer, ionic reaction at the particle interface, diffusion within the active materials, and phase-transfer reactions. As illustrated in Figure 6, in a battery electrode, the electronic conduction precedes the ionic reaction and diffusion processes, thereby regulating the ionic insertion process through electronic conduction. A high electrical conductivity of the electrode is thus essential for achieving optimal battery performance and a high electrical conductivity in the electrode is crucial for optimal battery performance. The characteristics and selection criteria of the basic battery components in Na-ion batteries are explained under subheadings in detail for further elaboration.

### 3.1. Anodes

**Figure 7:**
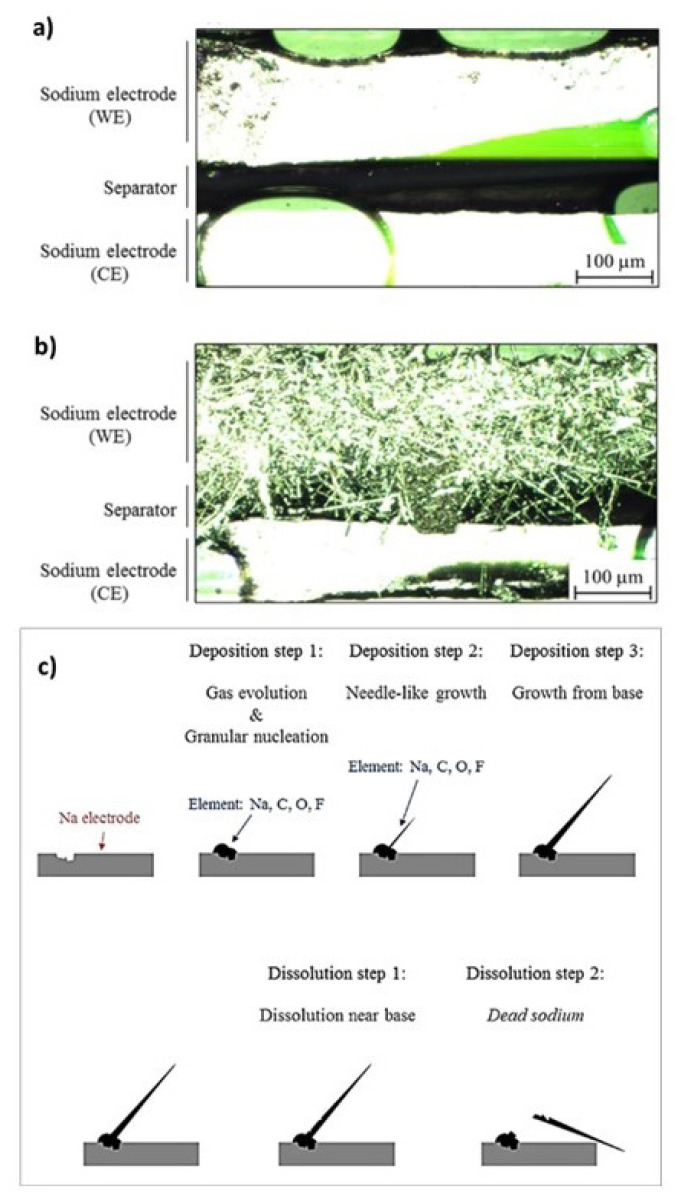
Cross-sectional views of a sodium electrode/separator/sodium electrode before (a) and after sodium deposition (b) together with schematic representation of electrochemical sodium deposition/dissolution behavior on a sodium electrode [28]. (Figure reprinted with permission.)

Anodes are among the essential components in which electrochemical reactions occur during battery operation. Typically, they consist of materials capable of efficiently performing intercalation and deintercalation Na^+^ ions during charging and discharging cycles. While sodium metal may not exhibit optimal characteristics as an anode material in Na-ion batteries, it remains frequently utilized in scientific investigations. As the energy density of a complete battery, comprising both cathode and anode, is directly linked to the average operational voltage, an anode featuring a lower potential is favored to enable a heightened average operational voltage. Nonetheless, the anode’s potential should not descend excessively, nearing, for instance, 0 V relative to the standard Na/Na^+^ reference electrode (this condition may lead to significant safety concerns due to the formation of sodium dendrites, which could trigger severe risks) [25]. Yui et al. investigated electrochemical sodium deposition/dissolution behavior by means of in situ light microscopy [28]. According to their findings, sodium deposition follows three steps, while two stages constitute the electrochemical sodium dissolving process, as shown in Figure 7. Batteries assembled using Na metal have a short cell lifetime because it easily undergoes oxidation, resulting in the degradation of the battery’s characteristics. The battery’s operating temperature made of Na anode materials is one of the other significant issues that should be considered. It is known that the melting temperature of Na (97.7 °C) itself may not directly dictate battery performance, but it is an important factor to consider in the design, operation, and safety considerations of Na-ion batteries, especially in relation to ambient temperature fluctuations and potential thermal events. When selecting anode materials, it is essential to prioritize materials with a low ionization potential and a substantial unit cell volume to facilitate effective ion intercalation and deintercalation processes [29,30].

Several materials have been explored and developed for Na-ion battery anodes for decades. Four main categories can be used to classify anode materials for Na-ion batteries, delineated by distinct electrochemical reaction mechanisms between the anodes and reversible sodium ions. These are insertion materials, conversion materials, alloying materials, and organic materials. However, in the context of Na^+^ ion storage mechanisms, the literature classifies materials into three categories: intercalation/insertion, alloying, and conversion. One of the best classification which has been used today has been explained in the review of Perveen et al. [26]. Potential anode materials encompass a variety of options, including organic compounds like Schiff bases, carbonyls, or quinone derivatives, as well as inorganic oxides such as TiO_2_, Na_2_Ti_3_O_7_ (intercalation), Fe_2_O_3_, Co_3_O_4_, or CuO (conversion); elements of group 14 and 15 (such as Sn, Sb, P, Bi, and Ge) that alloy with Na; and with carbonaceous materials [31]. Research on anode materials for Na-ion batteries has sometimes been categorized under three subtitles based on the reaction type: insertion reaction materials (such as carbon and titanium-based oxides), conversion reaction materials (such as transition metal oxides (TMOs) or transition metal sulfides (TMSs)), and alloying reaction materials (such as p-block elements) [32–35]. Moreover, composite anode materials for Na-ion batteries consist of a combination of different material groups according to the literature [36–40]. These composite materials are designed to harness the strengths of individual anode materials while mitigating their weaknesses, ultimately enhancing overall battery performance due to synergistic effects and a tailored design. This versatility in the design of anodes enables the optimization of various parameters to achieve desired performance metrics, such as energy density, power density, and safety.

**Figure 8:**
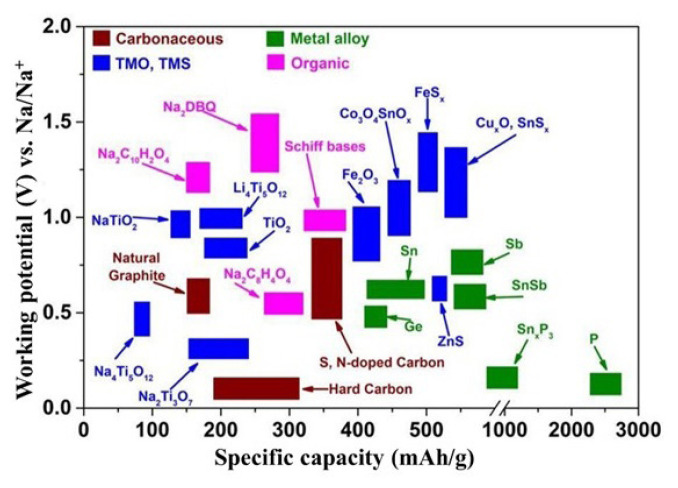
Specific capacity ranges of the Na-ion battery anodes [51]. (Figure reprinted with permission.)

Amongst the anode materials, carbon materials are of particular interest, as graphite is used as an anode for commercial Li-ion batteries. It is well known that lithium can readily undergo electrochemical intercalation within the graphite layers due to the layered structure of graphite exhibiting long-range order. Although graphite exhibits poor electrochemical sodium storage in Na-ion batteries due to its small interlayer distance (approximately 0.34 nm), hard carbons (nongraphitic or disordered carbons) have been found to successfully intercalate Na^+^ ions due to their structure [41,42]. The structure of hard carbons is characterized by randomly oriented, short turbostratic graphitic nanodomains featuring larger interlayer spacing compared to graphite. Additionally, it consists of curved and defective graphene nanosheets, along with closed pores interspersed among these disordered layers [43,44]. Based on the literature findings, carbons with various morphologies, including core-shell structures, spheres, fibers, and hollow structures, may be used as anodes for Na-ion batteries, and further capacity increases and long-term electrochemical stability may be achieved through approaches such as heteroatom doping [45–50]. Specific capacity ranges of different anode materials are shown in Figure 8.

### 3.2. Cathodes

Similarly with anodes, cathode electrode materials of Na-ion batteries play a critical role in the performance of the cell during charge and discharge cycles. Developing cathode materials that possess compatibility with the other components is crucial for advancing the technology. First, the cathode employed in Na-ion batteries must facilitate reversible accommodation of sodium cations at a voltage significantly exceeding 2 V positive to that of Na metal. This criterion is imperative since materials exhibiting low voltages (<2 V vs. Na) are appropriately categorized as anodes. The relationship between the voltage windows of the anode and cathode materials is crucial for the overall performance and safety of the system. This voltage window refers to the range of voltages over which the electrodes can effectively operate without causing detrimental side reactions or structural degradation. Ideally, the voltage windows of the electrodes should overlap to ensure that the cell operates within a suitable voltage range. This overlap enables effective charge transfer between the anode and cathode, maximizing the battery’s energy density, cycling stability, and overall performance. However, it is also essential to consider the potential mismatch between the anode and cathode voltage windows to prevent detrimental side reactions, such as electrolyte decomposition, voltage fade, or electrode degradation. Therefore, careful selection and optimization of both anode and cathode materials, along with electrolyte composition, are necessary to achieve a well-balanced and high-performance Na-ion system. Maximizing the energy density of Na-ion batteries entails strategies such as increasing the operational voltage of the cathode or reducing the operational potential of the anode. This can be further achieved by enhancing specific electrode capacities and, practically, manufacturing materials with densely packed particles, leading to high tap density. Cathodes exhibit superior performance when serving as host materials for sodium, wherein minimizing volume changes during sodium cycling is imperative for ensuring long-term battery cycling performance [52].

**Figure 9:**
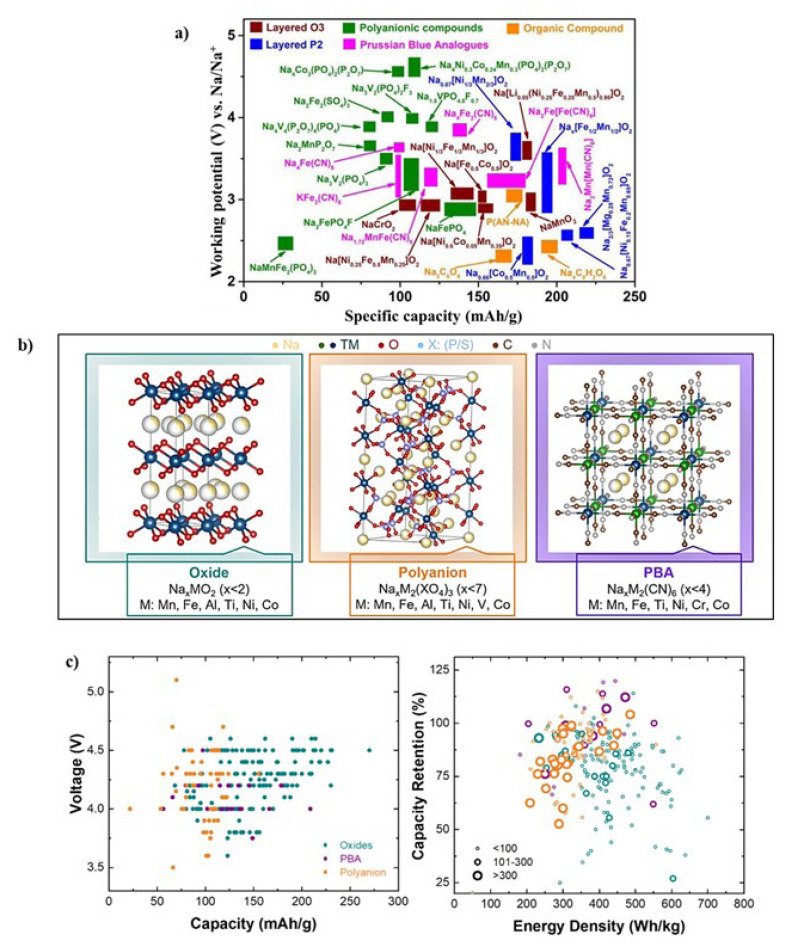
(a) Specific capacity ranges of the Na-ion battery anodes, (b) oxides, polyanions with NASICON as a representative, and Prussian blue analogues (PBAs), (c) upper cut-off voltage versus capacity and capacity retention versus energy density of cathode material classes [51,67]. (Figure reprinted with permission.)

Several families of materials have been thoroughly investigated for use as Na-ion cathodes, each offering unique advantages and challenges. The primary categories investigated as cathode materials encompass layered transition metal oxides, polyanionic compounds (including phosphates, fluorophosphates, mixed phosphates, etc.), Prussian blue derivatives, conversion materials (such as transition metal fluorides or oxyfluorides, sulfides, and selenides), and organic compounds (including conjugated carbonyls or redox-active polymers). Different families of materials explored as cathodes for Na-ion batteries with their strong and weak points are illustrated in Figure 9a and explained in detail by Goikolea et al. [1]. Each family of cathode materials presents unique opportunities and challenges in terms of energy density, cycling stability, rate capability, and cost-effectiveness. Research in this area continues to explore new materials, optimize existing ones, and address the limitations to enable the development of high-performance Na-ion batteries for various applications. A representative crystal structure of oxide, polyanion (NASICON), and Prussian blue analogues (PBAs) as the three main types of cathode materials for sodium-ion batteries are shown in Figure 9b. In the figure, the general formula for each type of material is given while M is the representative of the transition metals (TMs). To identify the best candidates for next-generation sustainable cathode structures, Sayahpour et al. compared experimental results of different cathode materials as given in Figure 9c [53]. Upper cut-off voltage vs. initial discharge specific capacity and capacity retention vs. energy density are illustrated in the figure, while the diameter of the circle correlates with the number of cycles. It should be noted that high energy densities often do not result in high-capacity retention or long-term cycling.

The transition metal oxide cathodes are attractive due to their structure stability and good electrochemical performance. In Li-ion batteries, layered oxide LiMO_2_ (M is transition metal Co, Mn, Fe, Ni, etc.) has garnered considerable attention owing to its outstanding electrochemical characteristics. Transition metal oxides, Na_x_MO_2_, are also a research hotspot. Sodium transition metal oxides primarily exhibit three distinct structural types: P2, P3, and O3, each characterized by its unique arrangement [54]. The number of oxide layer packs together with surrounding Na environment defines the subclass of layered transition metal oxides. In the nomenclature of these materials, the notation delineates the Na’s coordination environment (O for octahedral, P for prismatic), with the numerical value representing the count of unique interlayers encircled by distinct oxide layers. The inclusion of a prime symbol (′) signifies the presence of a distorted phase. The specific structure exhibits narrow ion diffusion pathways and an increased surface area during the processes of sodiation and desodiation, which provide benefits in stabilizing cycling capacity, despite the potential occurrence of multiple phase transformations [55,56].

Polyanionic compounds, formulated as A_x_M_y_[(XO_m_)^n−^]_z_, represent a wide group of materials including phosphates, pyrophosphates, mixed polyanionics, silicates, and sulfates. Today, the most promising ones seems to be vanadium containing NASICON-type phosphates, characterized by two different structures, Na_3_V_2_(PO_4_)_3_ and Na_3_V_2(_PO_4_)_2_F_3_ [57]. The NASICON group of materials are known to be sodium super ionic conductors that have outstanding ionic conductivity and strong structural stability even if the complete electrochemical extraction of Na^+^ ions from the NASICON framework is a complex task that has not yet been clearly documented. These NASICON-type cathodes are of significant interest to the Na-ion research community [58]. Generally, NASICON-based cathodes exhibit a stable crystal structure and feature 3D pathways for ion conduction. This 3D structure provides durable support during extended sodiation and desodiation, while the excellent thermal stability of these materials enhances their suitability for various electrochemical applications. Utilizing NASICON cathodes in full cells is feasible, contingent upon employing a counter electrode with a preferred NASICON-based crystal structure. Based on the redox couple, Rajagopalan et al. [59] classified these materials in three subgroups as V-based (V^x+/^V^y+^ redox couple-based materials), Mn-based (Mn^x+/^Mn^y+^-based multiple redox couple materials), and others. Na_3_V_2_(PO_4_)_3_ (known as NVP or SVP) is a well-studied cathode material for Na-ion batteries, known for its stable structure, good cycling performance, and promising electrochemical properties due to its structural stability and fast sodium ion conductivity. These characteristics arise from the influence of its three-dimensional framework and the P-O-V bonding. Nevertheless, the limited electrical conductivity of NVP restricts its capacity and rate performance [60]. Hence, effective methods to improve NVP’s electrical conductivity, such as applying carbon coatings (or creation of carbon composites) and introducing transition metal dopants are still under investigation [61,62]. Remarkably, NVP offers a high theoretical discharge capacity of 118 mAh/g involving the complete extraction of two Na^+^ ions between 2.5 V and 3.7 V versus Na^+^/Na. NVP also offers multiple valence states for sodiation/desodiation and shows minimal voltage polarization [63–66]. Due to its stable crystal structure and optimal operating voltage, Na_3_V_2_(PO_4_)_3_ (NVP) is regarded as a safer alternative to many other high-energy-density cathode materials. Additionally, NVP is considered environmentally benign compared to conventional cathodes. The abundance of its constituent elements not only reduces material costs but also enhances its potential scalability for commercial production—key advantages in meeting the growing demand for energy storage applications.

Another class of cathodes, PBAs, of formula Na_2_M[Fe(CN)_6_] (where M = Fe, Co, Mn, Ni, Cu, etc.), exhibit an open framework structure featuring plentiful redox-active sites and robust structural stability, rendering them suitable for application as cathodes in Na-ion batteries. As conversion-based cathodes, transition metal fluorides (MFx, where M = Fe, Ti, V, Co, Ni and Cu; and x = 2 or 3), oxyfluorides, sulfides (Fe_x_S_y_, Co_x_S_y_), selenides, or CuCl and CuCl_2_ are said to have high energy densities. When it comes to organics, a wide range of materials including conducting polymers, organosulfur compounds, organic radical compounds, and carbonyl compounds (PTCDA and disodium rhodizonate) may be used as cathodes. Despite being low cost and versatile in structure, organic anode materials suffer from dissolution in commercial electrolytes, which adversely affects their conductivity, working potential, and lifetime.

For a general comparison amongst the common classes of cathodes, it can be concluded that layered oxides have higher specific capacity and energy density compared to polyanions and PBAs but have shorter lifetimes. Layered oxides also have high electronic conductivity, enabling excellent electrochemical performance without coatings, unlike polyanions, which require carbon coating for good performance. Layer exfoliation and volume expansion in layered oxides cause capacity loss and short lifetimes, while strong covalent bonds in polyanions and PBAs support long-term cycling. The performance of oxides is influenced by their structure, Na content, and TMs. Most electrolytes use organic carbonate- or ether-based solvents, limiting the stable voltage to approximately 4.2–4.5 V vs. Na^+^/Na, which affects cathode material performance [67]. When it comes to mitigation of the challenges related to the insufficient structural integrity, inadequate storage stability, and sluggish kinetics of the cathodes, several strategies were proposed by Gupta et al. [68]. Morphology control, along with nanostructuring and nanodesign, surface modification, structural modulation, heteroatom doping, and sodium compensation strategies, may be applied to improve the electrochemical performance of cathode materials, depending on the nature and structure of the material.

### 3.3. Electrolytes

Electrolytes play a significant role in facilitating ion transport and enabling electrochemical reactions. In other words, they serve as carriers of ionic charges by proving a suitable medium for ionic conduction between cathode and anode. Ideally, the Na-salt/solvent combination is used as an electrolyte to provide low viscosity, high ionic conductivity, stability across a broad potential range, excellent thermal stability, and minimal toxicity. The first stated characteristics—high ionic conductivity and viscosity—are crucial primarily for electrochemical purposes. When cycling below room temperature or at high current rates, electrolytes with low ionic conductivities or high viscosities typically underperform. Additionally, it is preferred to use electrolytes that are resistant to degrading into side products. This is because uncontrolled SEI formation can cause reduced transport kinetics of ions, higher polarization, and a loss of charge carriers, all of which can speed up cell failure. Therefore, a commercially viable Na-ion battery must be capable of carrying out both of these functions, making the appropriate selection of electrolyte salt/solvent combination vital. Most importantly, the salt dissolved in the solvent should be chemically stable (without involvement of redox reactions, as well as reacting with the cell components). Regarding the solvent, it is preferable for it to be polar with a high dielectric constant, ensuring enhanced ionic mobility due to its low viscosity. Additionally, the solvent should exhibit inertness towards the charged surfaces of Na-ion batteries during cycling, alongside possessing a high boiling point and low melting point [69–71].

Commonly used salts, solvents, and additives in the electrolytes for Na-ion batteries are shown in Table 2. Most liquid electrolytes are blends of carbonate solvents like ethylene carbonate (EC) and diethyl carbonate (DEC), or ethers such as dimethoxyethane (DME) and tetraethylene glycol dimethyl ether (TEGDME). Carbonate solvent combinations offer a suitable balance of characteristics, like a wide electrochemical stability window and a good ionic conductivity. The salts that are frequently included in conventional electrolytes are sodium hexafluorophosphate (NaPF_6_), sodium bis(fluorosulfonyl)imide (NaFSI), sodium tetrafluoroborate (NaBF_4_), and sodium perchlorate (NaClO_4_). The salts that can be used in electrolytes exhibit high solubility attributed to the weak coordination between the sodium ions and the large anions, consequently facilitating high ionic conductivity across several organic solvents. Conventional salts for Na-ion batteries suffer a number of difficulties that are unrelated to their electrochemical performance in the Na-ion cell. The primary problematic aspects include price and safety, with NaPF_6_ and NaFSI being expensive, while NaBF_4_ shows weak coordination between the sodium ions and the large anions and explosive NaClO_4_. As a result, the newly designed alternative salts are more focused on reducing costs and enhancing safety without sacrificing ionic conductivity [14]. The most intriguing salts created for these kinds of uses are borate-based ones such as sodium bis(oxalato)borate (NaBOB) and its close cousins sodium bis(salicylo)borate (NaBSB) and sodium(salicylato benzenediol)borate (NaBDSB) [72,73]. Several noteworthy substitutes that are worth considering are Hückel-type anions such as 4,5-dicyano-2-(trifluoromethyl)imidazolate (NaTDI) and 4,5-dicyano-2-(pentafluoroethyl)imidazolate (NaPDI) [14]. Unfortunately, however, there is still a considerable danger of flammability in organic liquid electrolytes, which fosters the development of novel electrolyte systems, mostly aqueous and solid counterparts, each of which has unique properties. The aqueous system utilizes water as a solvent, offering intrinsic safety and environmental benefits; however, it is limited by a narrow electrochemical stability window of 1.23 V [74]. The selection of Na-salt, specifically altering the counter-anion to Na^+^, impacts both the chemical and electrochemical stability, along with the ionic conductivity. Typically, the anions serve as the electrolyte part primarily subjected to oxidation, establishing the upper voltage threshold for the electrochemical stability range. Concurrently, the strength of ion–ion interactions governs the quantity of charge carriers accessible, thereby influencing ionic conductivity [75].

Ionic liquids (ILs) have recently been examined as propitious electrolyte solvents due to their extreme structural diversity and exceptional characteristics. Therefore, ILs have garnered interest as potential electrolytes for Na-ion batteries due to their unique properties and some key advantages such as low volatility in addition to their improved cycling stability, rate capability, high conductivity, and excellent thermal stability [76–78]. Furthermore, ILs offer unique advantages since they consist of organic ions, enabling extensive structural variation and providing opportunities for property tuning [79]. The properties of ILs can be finely tuned by selecting specific cation and anion combinations and this tunability allows customization of the electrolyte to meet the requirements for the specific electrode combinations. It is also known that ILs often possess wide electrochemical windows, meaning they can withstand a wide range of voltages. Lahiri et al. and Lahiri and Endres explained the reason behind the improved electrochemical properties as ionic liquid electrolyte causing twin SEI layer formation, which led to better cycling capability [80,81].

In recent years, concentrated electrolytes, named “solvent-in-salt” electrolyte systems have also attracted attention due to their high thermal stability together with maintaining fast electrode kinetics. Concentrated electrolytes typically refer to solutions with high concentrations of electrolytes. It is thought that the formation mechanism and characteristics of SEI layers originating from concentrated electrolytes differ from those observed with traditional ones. Thus, concentrated electrolytes offer unique functionalities that are unattainable with conventional (i.e. diluted) electrolytes [82].

Research into concentrated electrolytes is ongoing, with efforts focused on addressing the challenges related to viscosity, electrode compatibility, and cost. By optimizing electrolyte formulations and exploring new salt/solvent combinations, concentrated electrolytes have the potential to enhance the performance, safety, and energy density of Na-ion batteries. Therefore, different salt and solvent mixtures have been tested to understand their effect on extremely concentrated electrolytes.

**Figure 10:**
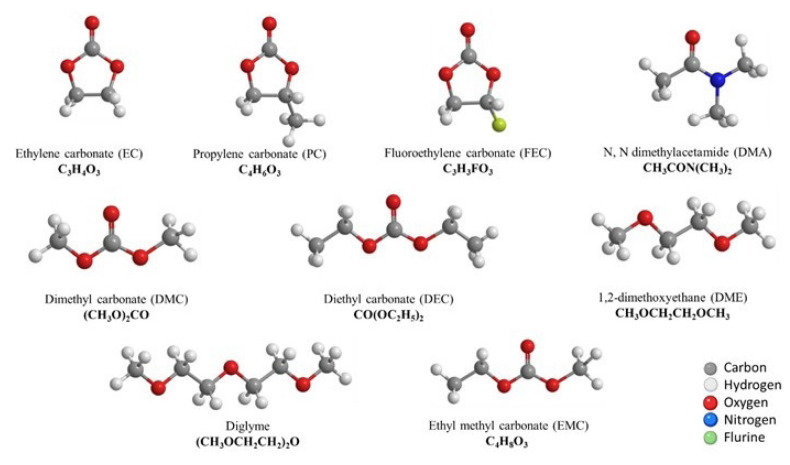
Some of the most widely used electrolyte solvents, cosolvents, and additives for Na-ion batteries [86]. (Figure reprinted with permission.)

It is worth mentioning that the development of solid-state electrolytes for Na-ion batteries is also attracting more attention since they do not require liquid electrolyte confinement, although they are currently unable to be employed for commercialization due to their inherent low ionic conductivity and the poor mechanical properties. Moreover, Na-ion conducting gel polymer electrolytes offer high ionic conductivity values and outstanding flexibility, making them one of the most potential options for the fabrication of flexible devices [83]. The gel matrix provides mechanical support to the electrolyte, reducing the likelihood of electrolyte leakage or structural damage, especially under mechanical stress or during battery operation. Through the encapsulation of liquid electrolytes within a solid polymer framework, such as poly(ethylene oxide) (PEO), poly(acrylonitrile) (PAN), poly(vinylidene fluoride) (PVDF), poly(methyl methacrylate) (PMMA), or poly(vinylidene fluoride-co-hexafluoropropylene) (PVdF-HFP), hybrid electrolyte systems can show the combined cohesive characteristics of solid and diffusive properties of liquid electrolytes. As a result, gel polymer electrolytes possess enhanced characteristics including wide temperature stability, favorable compatibility with electrodes, improved mechanical stability, high ionic conductivity, and mitigation of safety risks [84,85]. The chemical structures of several commonly used electrolyte solvents, cosolvents, and additives for Na-ion batteries are depicted in Figure 10.

Additives added to electrolytes in minor amounts have generally been shown to be beneficial for improving battery performance. There are a few key functions to be studied in relation to the purposes of additives. Additives are targeted to solve the issues concerning film formation, flame retardancy, and overcharge protection, together with functioning as salt stabilizers, wetting agents, and corrosion inhibitors for current collectors. There are many examples of additives for Na-ion batteries, but among all, FEC is the one that is used most; it is primarily utilized for its exceptional ability to enhance anode passivation. These additives, tailored to broaden the electrochemical stability range, undergo degradation preceding the electrolyte, forming passivation layers that effectively inhibit further reactions on both the anode and cathode.

### 3.4. Binder, separator, and current collectors

The binder is just as critical as the electrolyte; it is utilized for affixing the active electrode materials to the metal current collectors. An appropriate binder should be able to stabilize the electrode surface and prevent electrode deformation during cycling. Considering the existence of a reactive environment inside a Na-ion battery, the binder needs to endure both thermal and electrical conditions. The processability and the performance within the cell are crucial factors in the selection of the binder. When identifying alternative binders, three criteria, namely processability, chemical composition, and natural availability, should be considered for the assessment of their environmental impact. For example, processability implies processing using eco-friendly solvents like water or ethanol. In terms of environmental impact, fluorine-containing decomposition products of fluoropolymers have adverse effects and should be avoided by using more sustainable alternatives [87]. However, poly(vinylidene fluoride) (PVdF) is known to be the typical binder used because of its reasonable electrochemical stability and capacity to cling to the current collector between the electrodes. Some water-soluble binders, such as sodium alginate, (Na-alg), polyacrylic acid (PAA), sodium carboxymethyl cellulose (Na-CMC), and sodium polyacrylate (PANa), are regarded as potential replacements of PVdF to avoid toxic solvents during the slurry preparation stage [88]. In Table 3, the electrochemical performance of several binders used in different active material formulations was compared and the effects of active material, electrolyte, and cell configuration were highlighted [89–103]. It should be concluded that the performance of the binder is primarily determined by several key factors in the early research stage of Na-ion battery systems. In particular, compatibility of the binder with other components in the battery, such as the electrolyte and active materials, is crucial in addition to its adhesion strength. Furthermore, the binder should possess adequate mechanical strength and flexibility to accommodate volume changes in the electrode materials during cycling.

For Na-ion batteries, separators act as electronically insulating layers between electrodes to prevent internal short circuits. Therefore, separators must exhibit good Na-ion conductivity in electrolytes, along with insulating properties, high electrochemical and thermal stability, and sufficient mechanical strength [104]. Separators should readily wet in the electrolyte to create pathways for the migration of Na^+^ ions; thus, the wettability of the separator with the liquid electrolyte significantly affects battery performance [105]. In addition, the separator should be produced at a reasonable cost and with a flexible structure. Although the glass fiber separator is a typical separator used in research with the benefits of good thermal stability and abundant porosity, its major drawback is its mechanical strength. In addition, glass fibers are excessively thick; their standard thickness of about 300–500 μm may lead to a significant reduction in cell capacity per unit volume for the feasible design of a commercial the Na-ion battery [106]. It is also known that large pores (tens of micrometers in size) of glass fiber may cause internal short circuiting between electrodes that restrict their practical [107,108]. Therefore, strategies have been developed to identify alternative separators, coatings, and composite selective films. For this purpose, several fabrication and modification processes for thin films and membranes are also under investigation, including dip-coating, thermal polymerization, solution casting, and electrospinning [109–112].

## 4. Interphase formation on electrodes

**Figure 11:**
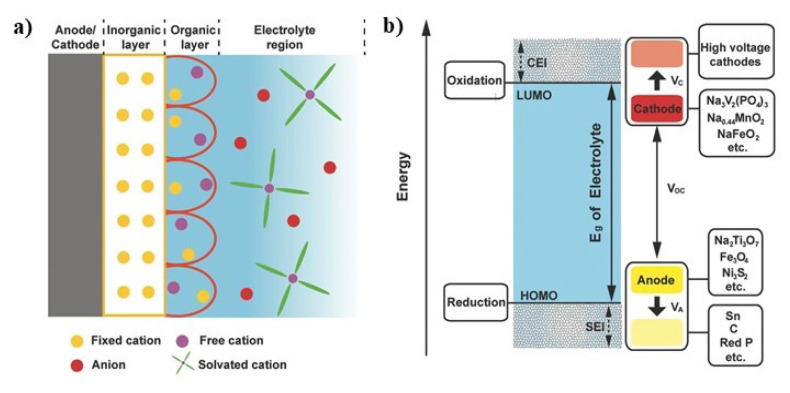
(a) Schematic illustration of an SEI and CEI together with Na^+^ ion transfer, (b) CEI and SEI formation under electrochemical reduction/oxidation conditions [113]. (Figure reprinted with permission.)

It is well known for Na-ion batteries that interphase formation together with morphology, thickness, and composition of the interphase considerably affects electrochemical performance. The interfaces between electrodes and electrolytes, referred to as solid–electrolyte interfaces (SEIs) on the anode surface or cathode–electrolyte interfaces (CEIs) on the cathode surface, are primarily influenced by the chemical and electrochemical interactions of sodium salts dissolved in aprotic solvent molecules. Maintaining a stable SEI on the anode surface and CEI on the cathode surface during initial cycles is critical for cell life. After the formation of SEI and CEI, the chemical and electrochemical reactivity and stability of the cell components will dictate the reversibility and rate of the battery. Occasionally, an interphase emerges at the boundary between the electrode (either cathode or anode) and the electrolyte during the decomposition of the latter, effectively inhibiting parasitic reactions and providing kinetic stabilization to the system. Figure 11 illustrates the electron energy levels relative to the anode, electrolyte, and cathode of a thermodynamically stable redox pair during electroreduction and electrooxidation processes. At the open-circuit condition, the battery operates at the open-circuit voltage (VOC), determined by the disparity in electrochemical potentials between the anode (μa) and the cathode (μc) [114]. Electronic movement ceases, whereas ionic motion (working ion) persists within the cell until equilibrium is achieved between μA and μC through the internal electric field of the electrodes. At this point, the cathode acquires positive polarity, and the anode obtains negative polarity, resulting in an open-circuit voltage (also referred to as the working voltage) of the battery. The working voltage is constrained by the electrochemical range of the electrolyte and the energy differential between the lowest unoccupied molecular orbital (LUMO) and the highest occupied molecular orbital (HOMO) determines the working voltage. Put differently, the stability range of the electrolyte corresponds to the disparity in energy levels between the LUMO and HOMO. Selection of the anode and cathode must ensure that μa is positioned below the LUMO while μc lies above the HOMO; otherwise, the electrolyte risks reduction on the anode or oxidation on the cathode. If the electrochemical potential of the cathode exceeds the HOMO, electrons will be extracted from the electrolyte, leading to electrolyte oxidation and subsequent formation of the CEI, thereby preventing further electrolyte oxidation. On the other hand, when the LUMO of the electrolyte is lower than the Fermi energy of the anode, reduction of electrolyte on the anode occurs to form an SEI layer as illustrated in Figure 11. The blue background in the figure indicates the electrochemical stable window (E_g_) of the Na electrolyte, while the red and yellow square indicators show the operating voltages of the cathode (V_C_) and anode (V_A_), respectively.

In comparison to SEI, CEI has received less attention since it is considered to have a less significant impact on the electrochemical performance of Na-ion batteries. In particular, most cathode materials, with the exception of high-voltage materials, are operated within the stable voltage window of an organic electrolyte (4.3 V), in which the CEI demonstrates only a minor contribution to the performance of the batteries [115]. Therefore, specific attention has been focused on SEI to achieve Na-ion batteries with long-term cyclic stability.

SEI concept was first introduced by Peled [116] and then accepted in battery science as the nature of electrodes and electrolyte is antecedent. It is important to visualize the SEI layer as an electrically insulating and ionically conducting interphase. SEI facilitates ion diffusion while impeding electron flow, thereby reducing overpotential and concentration polarization, and serving as a protective passivation layer to prevent further electrolyte decomposition in subsequent cycles. Moreover, the SEI layer allows the flow of electrochemically active particles; however, it increases the battery’s internal resistance, leading to capacity loss and impacting battery performance. The ideal SEI film should exhibit low electrical conductivity, along with excellent chemical, electrochemical, and mechanical stability and a high ion diffusion rate. Consequently, the SEI film effectively mitigates self-discharge, pulverization, and internal short circuits in batteries, while preventing damage caused by the solvent molecules and electrode materials cointercalating, thereby significantly enhancing battery cyclic stability [61]. As the SEI formation entails a loss of charge and electrolyte, its ideal completion occurs during the initial cycles and should persist stably throughout long-term cycling. Constant disintegration of the SEI causes it to undergo reconstruction in later cycles, resulting in inadequate coulombic efficiency, substantial irreversible capacity losses, and eventual battery death.

Due to the lower charge density and dissociation energy of sodium salts compared to lithium salts, the solubility of SEI components formed in Na-ion batteries generally surpasses that in Li-ion batteries, particularly in aqueous solutions. It has been established that capacity loss due to SEI dissolution is influenced by the electrolyte composition and electrode surface area. The mechanical properties of the SEI are primarily contingent upon formation conditions such as charge rate, electrolyte composition, and temperature. Furthermore, the solubility and oxidation of different substances influence SEI structure and porosity [86]. It is also known that formation of the SEI can be expedited through chemical modification, achieved by electrochemically reducing additives to chemically coat an organic film layer onto the electrode surface. Such SEI-forming improver additives can be either reduction-type or reaction-type. Reduction-type additives may include either a polymerizable monomer or a reducing agent, which may cause a stable SEI layer formation. To form a stable SEI, reaction-type additives serve as scavengers of radical anions or as complexes with the degradation products [117].

## 5. Challenges, opportunities, and the current research status of Na-ion batteries

Currently, there is great interest in the production of Na-ion batteries with high capacity and long-term cycle stability. However, optimizing the charge storage capacity of the electrodes, as well as the operating voltage, remains a challenging task to meet the expectations of future energy storage systems. Several approaches can be utilized to enable the application of new types of electrode materials [118,119]. For instance, dimension and morphology control, composite formation, doping, functionalization, and encapsulation strategies can improve the capacity of electrodes. Moreover, modification of electrolytes may also contribute to the formation of an interface layer on the surface and control the solubility of active materials and decomposition products.

These approaches address issues related to Na^+^ ion transport and aim to improve structural integrity, stabilize the surface reactions, and enhance the conductivity, and thermal stability of materials. However, specific attention should be paid to the type of materials and their crystal structures. Meanwhile, further research is needed to investigate the effects of interactions between the components. Additionally, studies should focus on the synthesis of alternative electrolytes and the modification of electrolytes to promote the formation of controlled passivation layers and to control the solubility of active materials and the formation of decomposition products. To gain more insight into the formation of the passivation layer, the focus of fundamental research should be on the nanocharacterization of these layers and mechanistic studies, although the progress made to date is remarkable. At this point, modern material characterization has relied heavily on in situ, ex situ, and in operando studies. These strategies enable researchers to investigate the relationships between structure and function in electrode materials or entire cell components. To support in-depth investigations on potential materials and designs, more experimental and theoretical investigations using cutting-edge characterization tools must be carried out to fully understand the reaction mechanisms during the sodiation/desodiation processes and their relationship with interfaces. Then further research should be extended to the system level including different cell combinations such as coin cell, pouch cell, and prismatic cell. Moreover, further long-term tests of different cell configurations with comparable data are urgently needed.

**Figure 12:**
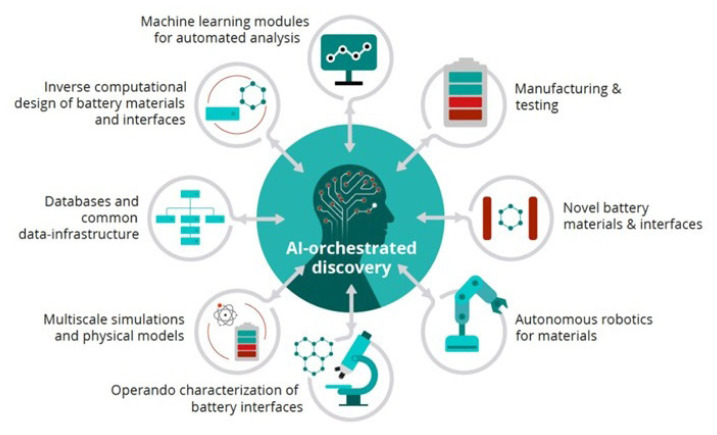
Key contribution of AI in the creation of a next-generation battery roadmap [120]. (Figure reprinted with permission.)

While it should be noted that the issues could have a game-changing effect on the performance of Na-ion batteries, manufacturing, scaling up, safety, and cost factors will ultimately determine commercial viability. For feasible industrial production, the whole supply and process chain should be systematically processed and analyzed in terms of materials and energy consumption to reduce the costs and production times of more sustainable Na-ion batteries. At this point, the definition of the structure-electrochemical property relationship of materials, as well as the integration of the current Li-ion production lines to Na-ion batteries, is a vital prerequisite. Recent modeling approaches using artificial intelligence (AI) also have great potential to identify interdependencies between several variables from material synthesis to process optimization. As emphasized in the BATTERY 2030+ Roadmap [120] and illustrated in Figure 12, advanced modeling tools can help with the design, development, and assessment of any novel Na-ion material and cell and feasible manufacturing procedure from several complementary approaches and technologies. In this way, not only will Na-ion battery characteristics such as capacity, lifetime, and operating limit be enhanced but also positive influences of these batteries on the economy and environment will be determined.

It should be noted that collaboration between academia, scientific research organizations, and businesses to expand the supply chain of Na-ion batteries both upstream and downstream is essential at this stage to enhance the overall competitiveness of the industry and facilitate wholesome and sustainable growth [121,122]. The most important thing to keep in mind is that extensive experience in the production of Li-ion batteries on an industrial scale will probably lead the way for Na-ion systems and hence the commercialization path for Na-ion batteries appears to be relatively straightforward.

## 6. Concluding remarks

This review provides a summary of Na-ion batteries, their main components, and recent developments. It highlights the need for novel battery materials and their significance in advancing Na-ion batteries for future generations. Research strategies focused on innovative functional materials will undoubtedly push the boundaries of cost, cycling stability, energy and power density, and safety, to meet the anticipated rapid expansion in energy storage demand for grid energy systems and transportation.

One of the most significant yet challenging issues is the selection, development, and production of commercially viable electrodes, electrolytes, and other essential components for designing effective cells, despite the substantial achievements already made. Importantly, Na-ion technology can leverage the advancements accomplished with Li-ion technology, which has the potential to accelerate process integration.

However, more focused research is critically needed for these next-generation systems due to the lack of fundamental knowledge regarding the electrochemical cell chemistry of Na-ion systems. In addition to advancing scientific understanding of battery performance, there is an urgent need to develop feasible manufacturing processes by leveraging insights gained from Li-ion production experience to effectively adapt to market demands.

## Figures and Tables

**Table 1:** Desired properties of battery components [27].

Cathode	Anode	Electrolyte
Nonreactive with electrolyte	Nonreactive with electrolyte	Nonreactive with electrodes
Good oxidizing agent	Good reducing agent	Should not conduct electricity
Appropriate operating voltage	High Coulombic efficiency	Superior ionic conductivity
Structural stability	Structural stability	Safe to handle
Cost effective	Cost effective	Cost effective
Ease of synthesis	Ease of synthesis	Properties should not change with temperature variations
Good conductivity	Good conductivity	

**Table 2:** Compositions of the Na-ion battery electrolytes [71].

Sodium salts	Solvents	Additives
NaPF_6_ NaClO_4_ NaFSI NaTFSI NaBOB NaBF_4_ NaDFOB Highly concentrated electrolytes	Esters (EC, PC, DM, etc.) Ethers (DME, DOL, etc.) Nitriles Sulfones Ionic liquids	Film formation additives (FEC, VC, etc.) Flame retardant additives (TMP, DMMP, PFPN, etc.) Overcharge protection additives (BP, etc.)

**Table 3:** Comparison of the effects of various conventional and novel binders on the electrochemical performance of Na-ion batteries.

Binder	Active material	Cell configuration	Highest specific capacity at C-rate (or at current density)	Highest capacity retention	Main findings about binder	Ref.
➢ **CMC** ➢ **PVdF**	Na_3_V_2_(PO_4_)_2_F_3_ as cathode	H/C and F/C	***With CMC:*** 75 mA h/g at 70 C	***With CMC:*** ≈40 mAh/g after 3500 cycles at 30 C (Capacity retention = 79%)	➢ Switching the binder from PVDF to CMC results in a capacity four times higher. ➢ CMC induces a solid permeable interfacial film, which reduces resistance and maintains electrode integrity. ➢ The CMC–Super P conductive network provides extended charge transfer pathways and a porous structure.	[89]
➢ **CMC** ➢ **PVdF**	SnO_2_/hybrid carbon nanocomposite as anode	H/C	***With CMC****: *688 mAh/g at 50 mA/g	***With CMC:*** 98 mAh/g after 500 cycles at 200 mA/g	➢ The EC/DEC electrolyte solvent and CMC binder work synergistically to enhance performance. ➢ Na-CMC, a cross-linked polymer, provides good adhesion and prevents electrode collapse during the volume expansion of metal oxides.	[90]
➢ **CMC** ➢ **PVdF**	Hard carbon (cellulose, lignin, chitin, and chitosan derived) as anodes	H/C	***With PVDF/Cellulose-derived carbon:*** 332 mAh/g at 0.1 C	***With PVDF/Cellulose-derived carbon:*** 50 cycles at 0.2 C with a capacity retention of 97%	➢ Carbon additives typically reduce initial Coulombic efficiency with a slightly greater impact on PVDF-based formulations compared to those with CMC. ➢ The binder’s impact on iCE is more significant with CMC than PVDF.	[91]
➢ **PVDF**	Hard carbon (chitin and chitosan derived) as anodes	H/C and F/C	***With PVDF/Chitin-derived carbon:*** 280 mAh/g at 0.1 C	***With PVDF/Chitin-derived carbon:*** ≈260 mAh/g after 50 cycles at 0.2 C	➢ Hard carbon is compatible with PVDF. ➢ Acid washing of electrode affects electrochemical reactions and polarization.	[92]
➢ **Na-alginate** ➢ **PVdF** ➢ **PEO**	MoS_2_ microflowers as anode	H/C and F/C	***With Na-alginate:*** 820 mAh/g at 0.1C	***With Na-alginate****:* 595 mAh/g after 50 cycles at 0.1 C	➢ MoS_2_ with Na-alginate enable efficient charge transfer due to a strong electron transport network. ➢ The stability of the MoS_2_ microflowers/alginate interfaces contributes to consistent capacity retention over long-term cycling.	[93]
➢ **Cross-linked Na-alginate/Graphene oxide (SA/GO)** ➢ **Na-alginate** ➢ **PVdF**	MoS_2_ as anode and Na_3_(VO)_2_(PO_4_)_2_F as cathode	H/C and F/C	***For Anode with SA/GO****:* 442 mAh/g at 0.1 A/g ***For cathode with SA/GO:*** 0.5 C is 116.8 mAh/g ***For full-cell:*** 83.2 mAh/g at 0.05 A/g	***For anode with SA/GO:*** 2803 mAh/g after 200 cycles at 0.1 A/g ***For cathode with SA/GO:*** 117.9 mA/g after 300 cycles at 0.5 C ***For full-cell:*** a capacity retention of 90% after 300 cycles at 0.1 A/g	➢ The SA-GO binder has a 3D network structure that forms a passivation layer on the electrode surface, preventing electrolyte decomposition and promoting uniform SEI and CEI. ➢ The SA/GO reduces Na^+^ diffusion impedance, enhances Na^+^ migration, and helps mitigate volume expansion, during long-term cycling.	[94]
➢ **PVdF** ➢ **Na-PAA**	N-doped carbon nanotube as anode	H/C	***With Na-PAA:*** 378 mAh/g at 0.2 C	***With Na-PAA:*** 175.5 mAh/g after 300 cycles at 0.8 C	➢ Na-PAA with moderate molecular weight enhances electrode capacity by forming a smooth, stable SEI layer and reducing internal resistance. ➢ Na-PAA is a promising alternative to PVDF binders.	[95]
➢ **PVdF** ➢ **PAA**	Sn and Pb and Si (as anodes)	H/C	***For Sn with PAA:*** 758 mAh/g at 50 mA/g	***For Sn with PAA:*** 500 mAh/g after 20 cycles at 50 mA/g	➢ Improvement of the surface passivation of Sn electrode can be achieved with the binder (PAA) and the electrolyte additive (FEC) for the acceptable sodiation cycling.	[96]
➢ **CMC/PAA** ➢ **PVdF** ➢ **CMC**	FeP as anode	H/C	***With PVdF:*** ≈764.7 mAh/g at 50 mA/g	***With CMC/PAA:*** 321 mAh/g after 60 cycles at 50 mA/g	➢ Combined CMC/PAA binder can form a cross-linked structure that has high tolerance for the internal mechanical stresses. ➢ FEC addition to the electrolyte and using CMC/PAA binder enhances cycle life.	[97]
➢ **PANa**	FeP_4_ as anode	H/C	1137 mAh/g at 89 mA/g	1023 mAh/g after 30 cycles at 89 mA/g	➢ The carbon additive enhances electrical conductivity and stabilizes the SEI layer due to the preformed SEI effect of the polyacrylate coating, improving capacity retention for slurries with PANa binder.	[98]
➢ **Gum arabic** ➢ **PVDF**	NiFe_2_O_4_ nanotubes (NFNTs) as anode	H/C	***With gum arabic:*** 680.5 mAh/g at 0.05 A/g	***With gum arabic:*** 320 mAh/g after 200 cycles at 0.05 A/g	➢ Electrodes made with gum arabic show better mechanical properties, tolerating volume expansion and stress changes during charging and discharging. ➢ The stable, thin SEI layer and improved surface electrical properties enhance electrochemical reaction kinetics.	[99]
➢ **Poly-γ-glutamate (PGluNa)** ➢ **PVdF** ➢ **CMC**	P2-Na_2/3_Ni_1/3_Mn_2/3_O_2_ (P2-NiMn) as cathode	H/C	***With PGluNa:*** ≈160 mAh/g at 0.05 C	***With PGluNa:*** ≈140 mAh/g after 50 cycles at 0.05 C	➢ The PGluNa electrodes exhibit better electrochemical performance than the PVdF electrodes. ➢ The PGluNa binder effectively prevents self-discharge by forming a thin layer on the P2-NiMn particles, which reduces electrolyte decomposition and corrosion. ➢ PGluNa electrodes have lower resistance during cycling, likely due to stronger adhesion and improved electrolyte penetration, preventing electrical isolation of P2-NiMn particles during volume changes.	[100]
➢ **SF polymerized dopamine (SFPDA)** ➢ **PVdF**	Na_3_V_2_(PO_4_)_3_ (NVP) and Na_3_V_2_(PO_4_)_2_F_3_ (NVPF) as cathodes	H/C and F/C	***For NVP with SFPDA:*** 109.3 mAh/g at 0.1 C ***For NVPF with SFPDA***: 124.2 mAh/g at 1 C	***For NVP with SFPDA:*** 95.5 mAh/g after 500 cycles at 0.5 C (capacity retention of 94.5%) ***For NVPF with SFPDA:*** 95.5 mAh/g after 500 cycles at 0.5 C (Capacity retention of 97.2%)	➢ SFPDA can construct a robust CEI layer to weaken the side reactions at the electrode–electrolyte interface and accelerate the Na^+^ transfer.	[101]
➢ **Sericin protein/poly (acrylic acid) (SP/PAA)** ➢ **PAA** ➢ **PVdF**	Na_4_Mn_2_Fe(PO_4_)_2_P_2_O_7_ (NMFPP) and Na_3_V_2_(PO_4_)_2_F_3_ (NVPF) as cathodes	H/C	***For NMFPP with SP/PAA:*** 91.4 mAh/ g at 0.2 C	***For NMFPP with SP/PAA:*** ≈60 mAh/ g after 100 cycles at 1 C (Capacity retention of 83.2%)	➢ The SP/PAA is electrochemically stable at high voltages and enhances ionic conductivity through sericin’s interaction with the ClO_4_^−^ anion of the electrolyte, and hence it resulted in additional sodium-migration paths with lower energy barriers. ➢ The strong bond between the binder and NMFPP protects the cathode from electrolyte corrosion, prevents Mn dissolution, stabilizes the crystal structure, and maintains electrode integrity during cycling.	[102]
➢ **Tetrabutylammonium (TBA) alginate** ➢ **Na-alginate** ➢ **PVdF**	Na_0.67_MnO_2_ as cathode	H/C	***With TBA-alginate:*** 164 mAh/g at 0.1 C	***With TBA-alginate:*** ≈60 mAh/g after 500 cycles at 1 C	➢ TBA alginate-based electrodes display improved electrochemical performance when compared to PVDF-based electrodes.	[103]

* H/C: half-cell, F/C: full cell.

## Footnotes

1 IEA (2021). Net Zero by 2050 - A Roadmap for the Global Energy Sector [online]. Website https://www.iea.org/reports/net-zero-by-2050 [accessed 30 September 2024].

2 BloombergNEF (2023). Electric Vehicle Outlook 2023 [online]. Website https://About.Bnef.Com/Electric-Vehicle-Outlook/ [accessed 30 September 2024].

3 Statistica (2023). Lithium-ion batteries - statistics & facts [online]. Website https://www.Statista.Com/Topics/2049/Lithium-Ion-Battery-Industry/#topicOverview [accessed 30 September 2024].

4 Mining.com (2023). Top 25 nations producing battery metals for the EV supply chain [online]. Website https://www.Mining.Com/Web/Ranked-Top-25-Nations-Producing-Battery-Metals-for-the-Ev-Supply-Chain [accessed 30 September 2024].

5 Statistica (2023). Lithium-ion batteries - statistics & facts [online]. Website https://Www.Statista.Com/Topics/2049/Lithium-Ion-Battery-Industry/#topicOverview [accessed 30 September 2024].

6 Mining.com (2023). Top 25 nations producing battery metals for the EV supply chain [online]. Website https://Www.Mining.Com/Web/Ranked-Top-25-Nations-Producing-Battery-Metals-for-the-Ev-Supply-Chain [accessed 30 September 2024].
